# Supplementary material for: Exploring white matter microstructure and the impact of antipsychotics in adolescent-onset psychosis
Source: PLoS One. 2020 May 29;15(5):e0233684. doi: 10.1371/journal.pone.0233684 (PMC7259775; doi:10.1371/journal.pone.0233684)
Supplement: S1 Fig — Extracted mean fractional anisotropy (FA), axial diffusivity (AD) and radial diffusivity (RD) values of significant FA (A) and AD clusters (B) identified with whole-brain TBSS. Data is presented as grey boxplots for early onset psychosis (EOP) patients and white boxplots for healthy controls (HC). ACR = anterior corona radiata, CC = corpus callosum, SLF = superior longitudinal fasciculus, PLIC = Posterior limb of the internal capsule, SFOF = superior fronto-occipital fasciculus. Note: Data is presented for descriptive purpose only. (DOCX) [file pone.0233684.s001.docx]

**
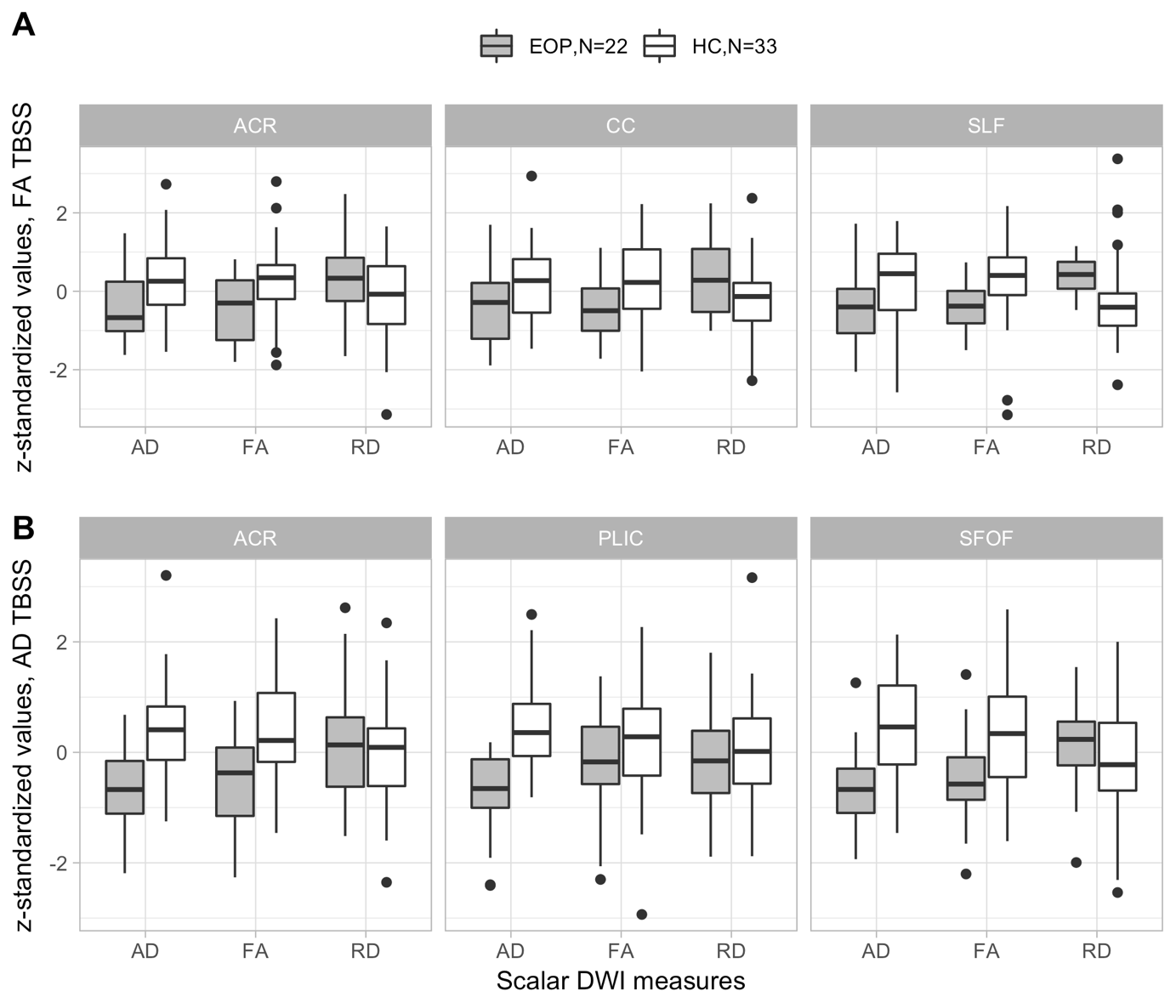
**

**S1 Fig. Extracted mean fractional anisotropy (FA), axial diffusivity (AD) and radial diffusivity (RD) values of significant FA (A) and AD clusters (B) identified with whole-brain TBSS.** Data is presented as grey boxplots for early onset psychosis (EOP) patients and white boxplots for healthy controls (HC). ACR = anterior corona radiata, CC = corpus callosum, SLF = superior longitudinal fasciculus, PLIC = Posterior limb of the internal capsule, SFOF = superior fronto-occipital fasciculus. Note: Data is presented for descriptive purpose only.
