# Supplementary material for: Exploring white matter microstructure and the impact of antipsychotics in adolescent-onset psychosis
Source: PLoS One. 2020 May 29;15(5):e0233684. doi: 10.1371/journal.pone.0233684 (PMC7259775; doi:10.1371/journal.pone.0233684)
Supplement: S2 Fig — Displayed are significant FWE-corrected TBSS results for FA (light blue–dark blue, p ≤ 0.05) and AD (red-yellow, p ≤ 0.05), contrasting EOP patients against healthy controls, overlaid on the study-specific mean FA image. The overlap of both DWI measures is depicted in green. Results shown underwent threshold-free cluster enhancement and are corrected for age and sex. CC = corpus callosum, ACR = anterior corona radiata, SLF = superior longitudinal fasciculus, R = right, L = left. Note: Data is presented for descriptive purpose only. (DOCX) [file pone.0233684.s002.docx]

**
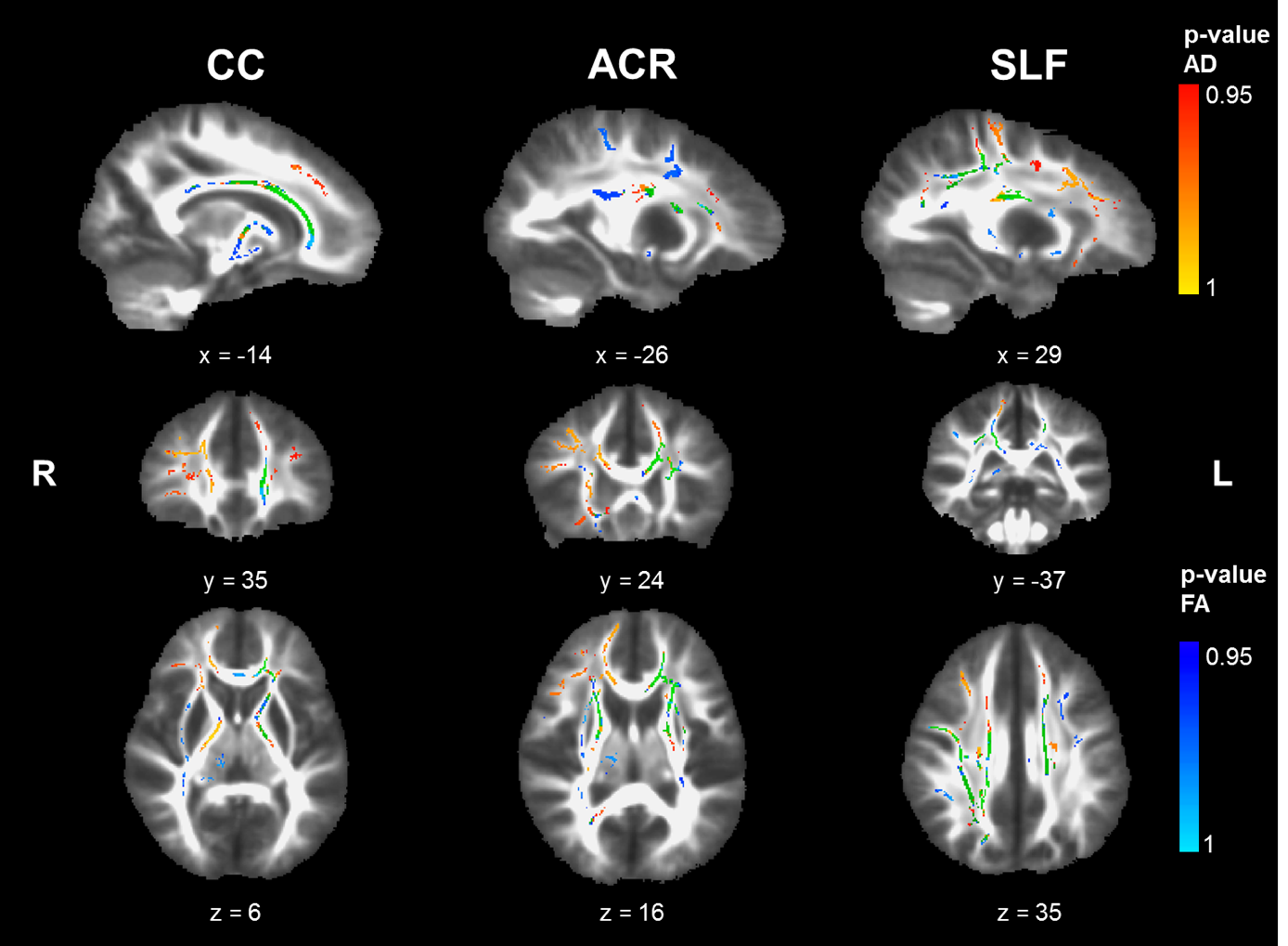
**

**S2 Fig. Lower fractional anisotropy (FA) and axial diffusivity (AD) in early onset psychosis (EOP) patients in comparison to healthy controls.** Displayed are significant FWE-corrected TBSS results for FA (light blue – dark blue, p ≤ 0.05) and AD (red-yellow, p ≤ 0.05), contrasting EOP patients against healthy controls, overlaid on the study-specific mean FA image. The overlap of both DWI measures is depicted in green. Results shown underwent threshold-free cluster enhancement and are corrected for age and sex. CC = corpus callosum, ACR = anterior corona radiata, SLF = superior longitudinal fasciculus, R = right, L = left. Note: Data is presented for descriptive purpose only.
