# Supplementary material for: Exploring white matter microstructure and the impact of antipsychotics in adolescent-onset psychosis
Source: PLoS One. 2020 May 29;15(5):e0233684. doi: 10.1371/journal.pone.0233684 (PMC7259775; doi:10.1371/journal.pone.0233684)
Supplement: S1 Table — (DOCX) [file pone.0233684.s003.docx]

**S1 Table|. Results of linear regression models with extracted mean fractional anisotropy (FA) and mean axial diffusivity (AD) values of significant TBSS clusters.**

| **Model *^1^** | **Estimate** | **S.E.** | **t value** | **p value** | **F** | **df** | **p** | ***Adj. R^2^*** |
| --- | --- | --- | --- | --- | --- | --- | --- | --- |
| **FA TBSS** |  |  |  |  |  |  |  |  |
| **ACR-IFOF** |  |  |  |  | 5.5 | 19 | 0.013 | 0.298 |
| DUI | 0.002 | 0.004 | 0.479 | 0.637 |  |  |  |  |
| AP | 0.048 | 0.016 | 2.991 | **0.008** |  |  |  |  |
| **SLF** |  |  |  |  |  |  |  |  |
| DUI | 0.009 | 0.008 | 1.190 | 0.249 | 0.9 | 19 | 0.434 | -0.012 |
| AP | 0.006 | 0.028 | 0.205 | 0.840 |  |  |  |  |
| **ACR** |  |  |  |  |  |  |  |  |
| DUI | 0.0001 | 0.006 | 0.020 | 0.985 | 1.0 | 19 | 0.388 | -0.001 |
| AP | 0.027 | 0.020 | 1.344 | 0.195 |  |  |  |  |
| **CC** |  |  |  |  |  |  |  |  |
| DUI | 0.001 | 0.005 | 0.210 | 0.836 | 0.02 | 19 | 0.977 | -0.103 |
| AP | -0.0003 | 0.016 | -0.016 | 0.987 |  |  |  |  |
| **AD** **TBSS** |  |  |  |  |  |  |  |  |
| **ACR-ATR** |  |  |  |  |  |  |  |  |
| DUI | -1.2e-05 | 5.1e-06 | -2.364 | **0.029** | 2.81 | 19 | 0.085 | 0.147 |
| AP | 1.6e-05 | 1.8e-05 | 0.855 | 0.403 |  |  |  |  |
| **PLIC** |  |  |  |  |  |  |  |  |
| DUI | -1.1e-06 | 5.1e-06 | -0.209 | 0.837 | 0.1 | 19 | 0.942 | -0.098 |
| AP | 6.1e-06 | 1.9e-05 | 0.324 | 0.750 |  |  |  |  |
| **SFOF** |  |  |  |  |  |  |  |  |
| DUI | -4.3e-06 | 5.3e-06 | -0.809 | 0.429 | 0.4 | 19 | 0.6801 | -0.061 |
| AP | -2.2e-06 | 1.9e-05 | -0.113 | 0.911 |  |  |  |  |

*^1^ Regional mean FA of significant clusters identified with Tract-Based Spatial Statistics (TBSS); S.E. = standard error, DUI = duration of illness & AP = antipsychotic use (coded yes (1)/no (0)); ACR-IFOF = anterior corona radiata, 16% inferior fronto-occipital fasciculus, SLF = superior longitudinal fasciculus, ACR = anterior corona radiata, CC = corpus callosum.
