# Supplementary material for: Exploring white matter microstructure and the impact of antipsychotics in adolescent-onset psychosis
Source: PLoS One. 2020 May 29;15(5):e0233684. doi: 10.1371/journal.pone.0233684 (PMC7259775; doi:10.1371/journal.pone.0233684)
Supplement: S2 Table — (DOCX) [file pone.0233684.s004.docx]

**S2 Table| Extended linear regression model for extracted mean fractional anisotropy (FA) of the left anterior corona radiata.**

| **Model *^1^** | **Estimate** | **S.E.** | **t value** | **p value** | **F** | **df** | **p** | ***Adj. R^2^*** |
| --- | --- | --- | --- | --- | --- | --- | --- | --- |
| **ACR-IFOF** |  |  |  |  | 8.78 | 14 | 6.03e-4 | 0.67 |
| AP | 0.045 | 0.017 | 2.624 | **0.020** |  |  |  |  |
| Handedness | 0.059 | 0.022 | 2.684 | **0.018** |  |  |  |  |
| Age | -0.556 | 0.156 | -3.556 | **0.003** |  |  |  |  |
| Age^2^ | 0.172 | 0.048 | 3.605 | **0.003** |  |  |  |  |
| Sex | -0.039 | 0.015 | -2.581 | **0.022** |  |  |  |  |

*^1^ Regional mean FA of significant cluster identified with Tract-Based Spatial Statistics; AP = Antipsychotic use (coded yes (1)/no (0)); ACR-IFOF = anterior corona radiata, 16% inferior fronto-occipital fasciculus
