# Supplementary material for: Exploring white matter microstructure and the impact of antipsychotics in adolescent-onset psychosis
Source: PLoS One. 2020 May 29;15(5):e0233684. doi: 10.1371/journal.pone.0233684 (PMC7259775; doi:10.1371/journal.pone.0233684)
Supplement: S3 Table — (DOCX) [file pone.0233684.s005.docx]

**S3 Table| White matter cluster of reduced axial anisotropy in early onset psychosis patients relative to healthy controls.**

|  |  |  |  | MNI coordinates in mm | | |  |
| --- | --- | --- | --- | --- | --- | --- | --- |
| **Cluster** | **Region*** | **Side** | **Voxels** | **X** | **Y** | **Z** | **t-values** |
| 3 | Anterior corona radiata (50%  Anterior thalamic radiation) | L | 620 | -23 | 18 | 15 | 4.93 |
| 2 | Posterior limb of internal capsule | R | 344 | 16 | -10 | 2 | 5.05 |
| 1 | Superior fronto-occipital fasciculus (18% Anterior thalamic radiation) | R | 138 | 21 | 12 | 22 | 5.17 |

* Johns Hopkins University International Consortium for Brain Mapping (JHU ICBM)-DTI-81 white matter atlas and JHU white matter tractography atlas (in brackets) were utilized to label significant clusters with specific tract names
