# Supplementary material for: Exploring white matter microstructure and the impact of antipsychotics in adolescent-onset psychosis
Source: PLoS One. 2020 May 29;15(5):e0233684. doi: 10.1371/journal.pone.0233684 (PMC7259775; doi:10.1371/journal.pone.0233684)
Supplement: Attachment — Submitted filename: ResponsesToReviewer.docx [file pone.0233684.s006.docx]

**Review PONE-D-20-03059**

**Reviewer #1:**

**Overall this manuscript is well written, rigorous and in a logical order. There are several remarks that need to be addressed. These are provided per section and require changes to be made in order to make the manuscript acceptable.**

**Response:** Thank you for your critical revision of the manuscript and comments. We have addressed the comments section-by-section below.

**Section 1: Introduction**

**a) The introduction is logical in understanding but should start with a statement of why this research is important. Please provide numbers on why there is a need to do research in psychotic disorder.**

**Response:** Thank you, and thank you for this comment. We have revised the beginning of the introduction section to highlight the importance of our research, as follows (see revised version without track changes for correct line number, introduction, page 3, line 40-52):

*“Psychotic disorders such as schizophrenia typically emerge during late adolescence or early adulthood, which debilitating consequences. Cases occurring earlier during adolescence are defined as early onset psychosis (EOP), with an age of onset before 18 years (1), and provide the exceptional opportunity to examine regional and disease-specific brain maturation after psychosis-onset.*

*EOP occurs rarely (2), affecting 0.05-0.5 % of the population (1), and shows a heterogeneous clinical presentation. Relative to adult patients with psychosis, EOP patients show worse long-term prognoses (3), and EOP constitutes one of the leading causes of lifetime disease burden for adolescents aged 15-19 years (4). While white matter microstructural alterations are well-established in adult onset psychosis (5), less is known about putative alterations in adolescent EOP. This is a critical research gap, as studying white matter abnormalities in adolescents with psychosis may provide important clues for understanding the pathophysiology of psychotic disorders. “*

**b) In the introduction the FA measure is introduced. This might measure some “integrity” of white matter. Introduce why it is needed to investigate RD and AD. Next explain shortly what is known about RD and AD in psychotic disorder. Generally, RD is increased in patients with psychotic disorder, while AD is unchanged compared to healthy controls. This leads to the hypotheses stated in the last part of the introduction.**

**Response:** Thank you for this comment. We revised the introduction of the scalar diffusion measures to better convey the importance of assessing RD and AD, next to FA. In the subsequent paragraph, we state what is known about FA, RD and AD in early onset psychosis.

The adjustments to the manuscript are as follows (introduction, page 3, line 53-62):

*“Diffusion weighted imaging (DWI) is a magnetic resonance imaging (MRI) technique that maps the Brownian movement of water molecules in the brain in vivo. As axon membranes and myelin provide natural barriers for water diffusion, DWI can be used to infer local tissue properties (6). Scalar measures of DWI include fractional anisotropy (FA), which characterizes the degree of diffusion directionality (6). A decrease in FA has been linked to white matter damage (7). Within each FA cluster, axial diffusion (AD) along the primary axis, and radial diffusion (RD) perpendicular to it, can be investigated to specify diffusion directionality in more detail (6). Both measures have been associated with different biological underpinnings. For instance, while increased RD has been associated with demyelination (8), decreased AD has been linked to axonal damage (6).”*

**c) It needs to be noted that there is critical developmental phase and several factors play a role in the aberrant development. Note that brain white matter develops till the age of 25 and that there is a difference between males and females. This might be related to a more frequent early onset psychosis in males.**

**Response:** We appreciate the input and included information concerning the age-and sex-related trajectories in white matter maturation in the revised manuscript (introduction, page 3-4, lines 62-66):

*“Scalar diffusion measures change across the lifespan. Throughout adolescence until early adulthood, FA increases and RD decreases in healthy individuals (9, 10), with males showing continuous white matter growth from childhood through early adulthood and females showing growth mainly during mid-adolescence (11). The trajectories of AD are less known (9, 10).”*

**Section 2: Methods and materials**

**a) A total of 27 patients and 40 controls were included which underwent MRI examination. Why is the control group larger compared to the patient group? Based on what information was this sample size determined?**

**Response:** We apologies for the confusion. The study sample was drawn from an ongoing longitudinal study, and all participants with the same diffusion-weighted imaging sequence were included. We rephrased this part to better convey this information (methods and materials, participants, page 6, lines 130-131):

*“A total of 67 participants (27 patients/40 controls) satisfied the inclusion criteria and were scanned with the same diffusion weighted imaging sequence. “*

**b) It was clearly described why participants were excluded in several steps in the study. What are strong motion artefacts in the DWI data? How was this quantified?**

**Response:** At first, the DWI data were visually inspected to identify obvious motion artefacts. Participants were excluded based on this visual inspection. Secondly, we extracted the motion estimates from the eddy current correction, and checked for average motion per volume higher than 2 standard deviation above mean. None of the remaining participants met this criterion.

We now included (methods and materials, participants, page 7, line 136-139):

*“Strong motion artifacts were identified by visual inspection and by calculating the average motion per volume higher than 2 standard deviation above mean per participants using the motion estimates from the eddy current correction (see section diffusion data analysis****)****.”*

**c) In the MRI data acquisition section, it is stated that “The diffusion imaging data was...”. The word data is plural and it should therefor read “The diffusion imaging data were...”.**

**Response:** We thank the reviewer for pointing it out, and changed it accordingly (methods and materials, MRI data acquisition, page 7, line 154).

**d) The MRI data are acquired with an anisotropic voxel size (2.5x1.875,1.875) which could have led to a less precise estimate of the diffusion tensor. This is an issue that needs to be discussed. The diffusion data analysis was done in a robust and fairly standard way.**

**Response:** We thank the reviewer for raising this point, and included a sentence acknowledging this limitation in the discussion section (discussion, page 22, lines 427-429):

*“DWI data were acquired at an in-plane resolution of 1.875 x 1.875 mm which could have led to a less precise estimate of the diffusion tensor. High resolution DWI should be used for future studies.”*

**e) In the manuscript the word gender was used, while the word sex more precisely describes the biological meaning of it. This needs to be replaced.**

**Response:** We thank the reviewer for pointing it out and replaced gender by the word sex accordingly (methods and materials, statistical analysis, page 9, line 182).

**f) In the analysis’s handedness was not taken into account as a covariate. Since the results report on left/right differences handedness needs to be added as a covariate.**

**Response:** We agree that handedness may influence bilateral results. However, in the current study we did not observe significant group differences in handedness between healthy controls and patients (see Table 1). Furthermore, handedness was evenly distributed between medicated and unmedicated patients with 10 right-hander and 1 left-hander in each group. The stratification of handedness between patient groups is now included in Table 2. To acknowledge a potential limitation of not including handedness as a covariate, we included the following sentence under limitations (page 21, line 426):

*“We did not adjust for handedness which could be associated with laterality differences.”*

**g) Significant findings from the TBSS analyses were extracted and further analyzed by extracting mean FA, AD and RD. These values were associated with illness duration. Why was this analysis conducted separately? This can also be done within TBSS and only in the patient group. Is it expected that there first is a group difference and next an association with duration on this difference between groups? It seems more logical to consider the illness duration within the patient group. Additionally, the illness duration might not be normally distributed within the group and needs to be reported (normal distribution test).**

**Response:** Thank you for this comment. We apologies for the confusion, and we realized that our description in the method/result section might have been misleading.

First, we run voxel-wise statistics contrasting case-control differences for FA, AD and RD, separately. We found significant lower FA in the left genu of the corpus callosum, the left anterior corona radiata (ACR), and the right superior longitudinal fasciculus (SLF) in EOP patients compared to healthy controls. Lower AD values were also found in the left ACR, right posterior limb of the internal capsule, and right superior fronto-occipital fasciculus. We did not find significant case-control differences for RD.

Second, we extracted patients’ mean FA from significant FA-TBSS clusters, and patients’ mean AD from significant AD-TBSS clusters to examine whether lower mean values in these brain areas in patients relative to controls were linked to patients’ duration of illness and antipsychotic medication status. Separate linear regressions were fitted for mean FA of significant FA-TBSS clusters and mean AD of significant AD-clusters. The results are summarized in **S1 Table.** Duration of illness was not significantly associated with mean FA in any of the significant FA-TBSS clusters. However, we found a significant negative association with mean AD in the left ACR (significant AD-TBSS cluster) and duration of illness. A finding most likely spurious as the regression model was not significant.

Third, we also extracted mean FA, AD, and RD for each of the significant FA-TBSS and AD-TBSS clusters for plotting purposes only (see **Fig 2 and S1 Fig**).

To better convey this information in the manuscript, we included the following changes:

Methods and materials (statistical analyses, page 8-9, lines 180 -195):

*“For contrasting case-control differences, we run voxel-wise statistics for FA, AD and RD, separately, using a nonparametric permutation-based approach (Randomise, implemented in FSL, 5000 permutations). Age and sex were entered as covariates. All covariates were demeaned. The cluster-forming significance threshold was set at p ≤ 0.01, after family-wise error correction for multiple comparisons using threshold-free cluster enhancement. We chose a conservative threshold to minimize type I errors and to account for the exploratory nature of the post hoc analyses.*

*For regions identified with TBSS (FA) and TBSS non-FA (RD/AD), we extracted the mean FA, RD and AD values of each participant at the set p-threshold of 0.01 for further analysis and illustration purposes.*

*Separate linear regression models were fitted to examine whether patients’ mean values of significant TBSS (FA) or significant TBSS non-FA (AD/RD) clusters, as dependent variables, were associated with duration of illness and antipsychotic medication status (coded as yes (1) / no (0)), as independent variables. The Cohen's d effect size was computed from the t-statistics for categorial variables, and using the partial correlation coefficient, r, for continuous variables (32).“*

Results (Linear regression analyses, page 15, lines 258-261):

*“For clusters identified with TBSS, we performed follow-up analyses to investigate whether patients’ lower FA and AD values in these brain regions were associated with duration of illness and antipsychotic treatment (S1 Table, available online). As we found no significant patient-control differences in RD using TBSS, no follow-up analyses using mean RD were performed. “*

The reviewer is also correct that duration of illness is not normally distributed, which is stated in table 2 and accounted for by displaying duration of illness as median [interquartile range] instead of mean ± standard deviation. For the linear regression models, diagnostic plots were generated and visually inspected to check for the assumption of homoscedasticity and normality. No violation was detected. Hence, for fitting linear models, a transformation of the duration of illness values was not needed.

**h) Clinical measures were associated with DWI measures within the patient group. This sample of 22 patients is very limited to test three DWI measures associated with three clinical measures. There are multiple tests in a small group and there is a risk of falsely reporting positive findings. Since a proper sample size calculation is missing, the results of this analyses should be interpreted with caution.**

**Response:** We agree with this comment, and have tried to interpreted the results with caution. Please also see our reply to comment 3. results, part d.

**Section 3: Results**

**a) Please share the statistical output from the TBSS analyses. Currently the manuscript has 2D images and the reader can be informed fully by providing the 3D images in MNI space. This allows for statistical pooling with other cohorts and meta-analyses. For example, this can be shared on FigShare. Note that these data are anonymous, because only statistical maps are shared.**

**Response:** We thank the reviewer for this valuable suggestion. We will deposit our statistical maps into a public repository, such as neurovault (https://neurovault.org), to make this data available to editors and peer-reviewers after publication.

**b) A description in text is missing for tables 1 and 2. Add a few short sentences on the sample characteristics and patient samples.**

**Response:** Thank you for spotting this. We now include the following sentences to describe table 1 and 2 (Results, demographics and clinical data, page 10, lines 205-214):

*“EOP patients did not differ significantly from controls in general demographic variables such as age, handedness and IQ (Table 1). In line with their clinical diagnosis, EOP patients showed higher impairment of general functioning evaluated with CGAS and exhibited significantly more depressive symptoms assessed with MFQ, relative to their healthy counterparts.*

*Within the patient group (Table 2), patients on antipsychotic medication were significantly older than unmedicated patients. Duration of untreated psychosis (DUP) was significantly longer in the unmedicated EOP patients in comparison to the medicated EOP patients. Other demographic and clinical variables did not differ significantly between medicated and unmedicated patients.”*

**c) In the results about the TBSS analyses it is reported that there are different mean values per group. It is unclear if these are mean values per group or that these values were not reported. If possible, report the mean values in text.**

**Response:** We apologies for the confusion. We realized that referring to mean FA and mean AD for the TBSS analyses might be misleading, and have now removed the word “mean” from the TBSS analysis section and were otherwise applicable. For the linear regression analyses, we extracted individual patient’s mean FA or mean AD values for each brain region showing significant case-control differences in the TBSS analysis. Minimum, maximum, median, first quartile and third quartile for each group (i.e. controls, medicated patients and unmedicated patients) FA, AD and RD of significant TBSS cluster are depicted in **Fig 2 and S1 Fig**.

We revised the following sentence in the method section to better convey that individuals mean values were extract (statistical analyses, page 9, line 187-189):

*“For regions identified with TBSS (FA) and TBSS non-FA (RD/AD), we extracted the mean FA, RD and AD values of each participant at the set p-threshold of 0.01 for further analysis and illustration purposes.”*

**d) The comparison between medicated and unmedicated EOP patients is fairly week due to the fact that both groups only have 11 individuals. Is there enough power to detect small differences in DWI measures?**

**Response:** We agree with the reviewer that the sample size is a limitation of the current study. We acknowledged this confound in the abstract and the discussion, and pointed out that a replication is warranted. Small samples sizes are, unfortunately, an inherent problem in the field of adolescence psychosis research, due to rare occurrence, and neuroimaging studies are sparse (reviewed by Tamnes and Agartz, 2016, J Am Acad Child Adolesc Psychiatry). However, due to the rare occurrence and the difficulties in recruitment, the current EOP sample is unique: The sample is well characterized, has a balanced number of medicated and unmedicated patients, and all participants were scanned on the same scanner. Unfortunately, though, a prior power analysis was not possible due to a lack of previous studies reporting effect sizes for the tentative effect of antipsychotic medication on white matter microstructure in medicated vs. unmedicated adolescents with psychosis.

We further emphasize the sample limitation in the discussion, now stating:

Discussion, Page 21, lines 414-417: *“The results of the current study should be considered in the context of several limitations. The cross-sectional nature of the current study precludes the assessment of developmental effects over time. The small sample size implies that the results should be interpreted with caution, and that findings require replication in a larger sample.”*

Discussion, Page 22-23, lines 443-447: *“Building on our tentative results, longitudinal studies with larger samples sizes in combination with clinical, genetic and neurocognitive measures are warranted to delineate heritability, affected brain regions, antipsychotic medication effects, and white matter microstructural differences over time.”*

**Section 4: Discussion**

**a) First start with a short recap of the main findings and next add previous findings.**

**Response:** Thank you for noting this. We have now added the following sentences to discussion to provide a short summary of the main findings (discussion, page 17, lines 299-303):

*“EOP patients showed significantly lower FA and AD in largely overlapping areas, particularly in left ACR, relative to healthy controls. In patients, mean FA of the left ACR was significantly associated with antipsychotic medication status, showing higher FA values in medicated compared to unmedicated EOP patients. Indices of white matter microstructure in patients were not associated with other clinical characteristics.”*

**b) In line 311 it is stated that a prediction was made. With the available data prediction is impossible. This needs to be rephrased.**

**Response:** We agree with the reviewer that the word “prediction” is not suitable and rephrased the sentence as follows (discussion, page 18, lines 321-323):

*“We found that FA values in the left ACR were significantly associated with antipsychotic medication status, showing higher FA values in medicated relative to unmedicated EOP patients.”*

**c) In line 315 and 316 a “This lack of significant associations” was described. This needs to be rephrased as “This null-finding”. It is important to report null-findings a bit more positive.**

**Response:** We agree and rephrased the sentence accordingly (discussion, page 18, line 333-335):

*“These null-findings are in line with previous studies in EOP patients (12, 18-21), and studies in adult-onset psychosis (5, 44).”*

**d) The statement that medication leads to an increase in FA detected by DWI is an over-interpretation of the results. First, there is no causality in the findings and therefor the word “leading” in line 339 needs to be replaced. Furthermore, a sharp note needs to be made on the small sample size in this part of the discussion. Readers might interpret this part of the discussion without taking note of the limited sample size behind it. This is at maximum an exploratory part of the study.**

**Response:** We appreciate the reviewer's input. We are aware of the speculative nature of our argument which we try to convey by using phrases such as “one might speculate”. We replaced the word “leading” to avoid confusion and added another sentence to highlight the limitation of the current sample size (discussion, page 19, lines 359-360).

*“In the current study, medicated EOP patients were treated with either received aripiprazole, quetiapine or risperidone. One might speculate that use of second-generation antipsychotics early in the disease process might affect white matter microstructure by remediating oligodendroglial dysfunction, reflected by an increase in FA detected by DWI. However, given the small sample size of the current study, no firm conclusions can be drawn.”*

**e) The sample size limitation is shortly discussed, without mentioning anything about an independent sample size calculation that would be needed. As this is post-hoc, nothing can be done about the results and using a p-value threshold at < 0.01 was already required for the multiple tests (for FA, AD and RD) that were performed. The conclusion is logically following based on the methods and results presented in the manuscript.**

**Response:** We agree with the reviewer, and refer to our answer to the reviewer’s previous comment (results, d).

**Reviewer #2:**

**In this study the authors used diffusion weighted imaging to examine white matter structure in people with early onset psychosis, and the effects of current antipsychotic use on WM. In this small sample, they found reduced FA in the EOP group, and WM differences in people on vs. off antipsychotic medications at testing, with the medicated group showing higher FA than the unmedicated group. Ascertaining EOP participants on and off medications is challenging, and investigation of both EOP generally and medication effects on WM specifically are both valuable. Several methodological notes are offered.**

**Response:** Thank you for the accurate summary and for your comments. We have addressed them below.

1. **This is obviously a very small sample, especially when dividing the EOP group into medicated and unmedicated subsamples. Effect sizes and confidence intervals should be reported for all main analyses, and findings should be interpreted as tentative.**

**Response:** We agree with the reviewer that the results are tentative, and the sample size is small, and we have tried to emphasize this further in the abstract and the discussion (see also reply to Reviewer 1, section 3. Results, part d). Additionally, we have upon your request included Cohen’s *d* effect size and the 95% confidence intervals for the significant main findings as follows (see revised version without track changes for correct line number):

Methods and materials (page 9, lines 193-195): *“The Cohen's d effect size was computed from the t-statistics for categorial variables, and using the partial correlation coefficient, r, for continuous variables (33).”*

Results (page 15, lines 262-264): “*Duration of illness was not significantly associated with mean FA in any of the clusters, but we observed a significant negative association with mean AD in the left ACR (d = -0.48, 95% CI [-1.67, 0.74], p = 0.029).”*

Results (page 15, lines 267-269): *“Exposure to antipsychotic medication was significantly associated with mean FA values in the left ACR (d = 1.37, 95% CI [0.01, 2.68], p = 0.008), showing higher mean FA in medicated relative to the unmedicated patients (Fig. 2).”*

2. **The medication-related analyses were exploratory with no ad-hoc hypotheses; this obviously has implications for interpretation, and (again) conclusions should be tentative. Oddly, in the discussion the authors imply that the findings make sense based on the literature, so it is puzzling that they do not make a priori hypotheses based on this literature.**

**Response:** Thank you for these comments. Due to a lack of previous findings in early onset psychosis in adolescent patients, the medication-related analyses were exploratory, and no clear hypothesis was stated. After analyzing the results, we found an association between FA values in the left ACR and medication status and discussed this new finding in light of animal studies and randomized trials in adults to better understand the potentially mechanisms underlying this tentative result. This discussion is intended to derive working hypotheses for future studies. To better convey the latter, we concluded the discussion as follows (discussion page 22-23, lines 441-447):

*“Understanding the significance of white matter abnormalities in the left ACR in adolescents with EOP and the putative effect of antipsychotic medication, may help to phenotype the disease and to improve treatment regimes. Building on our tentative results, longitudinal studies with larger samples sizes in combination with clinical, genetic and neurocognitive measures are warranted to delineate heritability, affected brain regions, antipsychotic medication effects, and white matter microstructural differences over time.”*

3. **The title focus seems a bit odd, as the stated aims in the manuscript were to 1) look at WM scalar measures in EOP and then 2) exploratory analysis of medication effects. The title (which makes medication effects central) should reflect the actual aims of the paper.**

**Response:** The reviewer is correct that the aim of the paper was two-fold and that the antipsychotic medication-related analyses were exploratory due to the lack of previous studies reporting medication effects in EOP. Currently, the title is supposed to reflect the main findings of the paper. However, we agree that a broader title could have be chosen and we have now changed the title to **“Exploring white matter microstructure and the impact of antipsychotics in adolescent-onset psychosis.”** which we believe reflects the manuscript better.

4. **While the FA findings were hypothesized, the authors found no difference in RD (contrary to hypothesis), and decrease AD (not hypothesized). Again, in a very small sample it should be noted that these were not expected findings and await replication.**

**Response:** Thank you, we agree with the reviewer, and have rephrased the following part of the discussion (page 20, lines 371-375):

*“Here, we found a widespread decrease of AD on a whole brain level, indicative of axonal damage, but there were no changes in RD, relative to healthy controls. These findings are not in line with our a priori hypothesis of increased RD and unchanged AD, and previous work by Lagopoulos and colleagues, reporting decreased FA in the left ACR driven by increases in RD (16). “*

5. **Though not statistically significant (likely due to small sample size), the off medication group had higher symptom severity than the on medication group. How was clinical severity controlled in the analyses?**

**Response:** The reviewer pointed out correctly, that symptom severity did not differ significantly between medicated and un-medicated patients. Symptom severity in patients was assessed using the Children’s Global Assessment Scale (group difference, p = 0.580), Mood and Feelings Questionnaire (p = 0.520) and Positive and Negative Symptom Scale (positive symptoms: p = 0.257; negative symptoms: p = 0.218; general symptoms: p = 0.489). While the mean values might look different, the range and statistical analyses indicates no difference between groups (see **Table 2**). Further, given the limited sample, it would be difficult to add additional covariates. However, we acknowledge that symptom severity likely determines whether patients are medicated or not, and we have now included the following sentence in the discussion to account for this dependency (page 21, lines 417-419):

*“While symptom severity did not differ between medicated and unmedicated patients at time of scan, prior symptom severity likely influenced the initiation of antipsychotic treatment, which may have influenced our results.”*

6. **Increased FA was reported in medicated compared to unmedicated patients; however, no association with CPZ load was found (which is a bit confusing). The authors attempt to address this in the discussion, but I think some degree of caution is needed here.**

**Response:** We see why it might be unexpected and try to account for it in the discussion as follows (page 18, lines 336-342):

*“Besides the high Cohen’s d effect size estimate, we found no significant association of regional FA with either current or cumulative antipsychotic exposure. These null-findings are in line with previous studies in EOP patients (12, 18-21), and studies in adult-onset psychosis (5, 44). For instance, studying white matter microstructure in 1,963 patients with schizophrenia, Kelly and colleagues found no significant effect of medication dosage (5). Hence, the usage of antipsychotic medication rather than the actual dose might induce the observed changes in white matter microstructure.”*

7. **The z-standardized values of AD in the patient groups (but not controls) are very highly skewed, as is the “off antipsychotic” group FA data. What statistical procedures were included to account for this issue?**

**Response:** We thank the reviewer for raising this important point. We addressed this concern by generating diagnostic plots for all fitted linear regression models to check for the assumption of homoscedasticity and normality of the residuals. As no violations of these vital assumptions for linear models were detected, no further correction was necessary.

8. **Some minor English language editing would be useful.**

**Response:** Thank you for this comment. We have thoroughly reviewed the manuscript and hope that the English language is improved. We have asked a native English speaker to read the manuscript.
