# Supplementary material for: Exploring white matter microstructure and the impact of antipsychotics in adolescent-onset psychosis
Source: PLoS One. 2020 May 29;15(5):e0233684. doi: 10.1371/journal.pone.0233684 (PMC7259775; doi:10.1371/journal.pone.0233684)
Supplement: Attachment — Submitted filename: ResponsestoReviewer2_final.docx [file pone.0233684.s007.docx]

**Review PONE-D-20-03059 R1**

**Reviewer #1:**

The manuscript has greatly improved with the implementation of the changes. Most of the comments were correctly realized. A few items remain;

**Response:** We thank the reviewer for the positive feedback and the additional comments. We have addressed the comments section-by-section below.

**Section 2: Methods**

**a.) In the methods section indeed it was clarified that 67 participants were recruited within the context of a larger longitudinal study. It still remains unclear why the healthy controls were oversampled compared to the patients. The comparison 27 patients versus 40 controls needs to be clarified. Was the expected effect size in patients almost twice the amount as in the controls? Why was this not an equal comparison of 27 patients with 27 controls? Also, perhaps the controls could have been matched with the patients based on age, sex and handedness.**

**Response:** We thank the reviewer for pointing this out. We agree that matching can be a valuable approach to increase the robustness of typical parametric tests such as t-tests when the patient sample is moderately powered. However, in our experience we have found matching approaches based on age and sex to be restrictive, and from a statistical point of view imprecise when the patient sample is small. A bigger control sample provides a closer estimate of the control variance and is more representative of the general healthy population. To ensure a representative adolescent control sample, we decided to include a larger control group. We included the following sentence in the manuscript to better convey this decision:

*“The larger control group was included in the study to provide a closer estimate of the control variance in adolescent populations.”*

**b.) The point about the anisotropic voxel size was partially addressed. There are limitations to the tensor fitting model and an anisotropic voxel size can influence the outcome measures. This point needs to be explained as a limitation to the current study. See DOI: 10.1016/j.neuroimage.2012.06.081 for more detail on this topic.**

**Response:** We thank the reviewer for providing this important reference. We included the provided reference as follows:

*“DWI data were acquired at an in-plane resolution of 1.875 x 1.875 mm which could result in* *less precise estimate of the diffusion tensor and the subsequent scalar diffusion measures (60). We acknowledge that the rather large anisotropic voxel size in the current study may have influenced the results, and that higher resolution DWI should be used for future studies.”*

**c.) It was decided not to include handedness as a covariate in the analysis. The authors agree that handedness may influence the bilateral results. Indeed there were no significant group differences on this variable, which is reassuring. Though, when assessing more detail in the comparison between the on medication and off medication patient groups it might be an issue. Most patients were right-handed which implies that the white matter in the right hemisphere is more developed. Therefore, findings might point to changes in the associations with white matter tracts in the right hemisphere. This could go either direction as there might be an interaction with medication use. In order to get an indication of how handedness might influence the findings, it is encouraged to include a sensitivity analysis. This can be done by extending the linear regression analyses on the TBSS clusters. It is fine to add this to the supplementary materials and shortly mention it in the results. In this way the main analyses can remain as it is and the sensitivity analysis can be added to inform about the influence of handedness on the results.**

**Response:** We thank the reviewer for the suggestion to include a sensitivity analysis. We extended the suggested sensitivity analysis by also including age, age^2^ and sex as other potentially confounding variables. We included the results of the sensitivity analysis in the supplementary materials (Table S2) and we describe the sensitivity analysis and its main results in the manuscript as follows:

*“To further assess the specificity of the association between antipsychotic medication and mean FA in the left ACR, we fitted an additional linear model that accounted for handedness, age, age^2^ and sex as potential confounds (S2 Table, available online). Age^2^ was included to account for putative non-linear effects of age on FA. The effect of antipsychotic medication on mean FA of the left ACR remained after adjusting for additional covariates (d = 1.40, 95% CI [-0.04, 2.78], p = 0.020) .”*

**d.) The comment on the sample size is addressed by stating that there is a lack of previous studies, and indeed there are limitations in this research field. This contradicts with the introduction part; “So far, studies in EOP patients do not indicate an impact of either current (18-20) or cumulative antipsychotic exposure (12, 19, 21) on scalar DWI measures. “. The studies in references 12, 18-21 do report on the association between antipsychotic medication use and DWI measures. To at least have an indication of the expected effect, an estimated effect size should be calculated based on the available data.**

**Response:** The reviewer is correct that reference 12, 18-21 indeed explore the effects of CPZ on white matter microstructure, yet none of these studies included unmedicated patients and most studies did region-of-interest instead of whole brain analyses (Moran et al. 2014, Schizophrenia Bulletin, Kumra et al. 2005, J. Am. Acad. Child Adolesc. Psychiatry, Kyriakopoulos et al. 2009, Br J Psychiatry). Hence, an effect size analysis based on this limited data will unfortunately be biased and is not going to aid the current study.

In the current manuscript, we acknowledge the lack of previous studies and state that the antipsychotic medication related analyses are exploratory. We chose a highly conservative threshold for FA to minimize type I errors and to account for the exploratory nature of the *post hoc* analyses. However, we agree with the reviewer that the introduction might be misleading and not clearly reflecting the previous findings and the aim of the current study. We therefore revised the introduction as follows:

*“Studies investigating white matter microstructure in EOP mainly focus on case-control differences, either reporting antipsychotic effects as secondary findings or using antipsychotic medication status as a covariate of no interest. So far, studies in EOP patients do not indicate an impact of either current (18-20) or cumulative antipsychotic exposure (12, 19, 21) on regional scalar DWI measures. The absence of an antipsychotic dosage effect in EOP patients could reflect small sample sizes and young patients with shorter medication histories. However, large-scale cohort studies in adult-onset schizophrenia also do not find an effect of medication dosage on white-matter microstructure (5, 22). Hence, medication dosage might not be a driving factor for potential effects of antipsychotic medication on the brain. So far, studies in EOP did not include unmedicated patients, which limits the ability to disentangle medication-mediated from disease-related effects on brain structure. Thus, zeroing in on unmedicated relative to medicated EOP patients provides the opportunity to investigate the impact of antipsychotic medication on white matter microstructure early in disease progression.”*

**e.) It is reassuring to read that there is an intention to share the data via NeuroVault. Would it be possible to have the link to NeuroVault integrated in the manuscript, before publication? Otherwise readers still need to search for the link after publication**.

**Response:** We thank the reviewer for raising this valid point. We will make sure that the link will be included in the manuscript after our work is accepted for publication, during authors-proof. This way, the link will be tied to the publication and the reader does not need to search for the link.

To ensure the reviewer that the statistical maps will in fact be released upon publication, we have added paragraph regarding this to the manuscript (prior to acknowledgements). We now include the following statement.

*“****Data availability***

*We made use of publicly available resources for processing the DWI data and for conducting the statistical analyses. Our clinical data is not publicly available, but we have released the statistical maps on NeuroVault (link to be inserted during author proofs).”*
